# The implementation of a Nationally Enhanced Service incentive for weight management: A longitudinal qualitative study of the perceptions and experiences of UK primary care staff on weight management using normalisation process theory

**DOI:** 10.1111/cob.70020

**Published:** 2025-04-29

**Authors:** Jack B. Joyce, Anisa Hajizadeh, Rachna Begh, Kate Jolly, Susan A. Jebb, Paul Aveyard

**Affiliations:** ^1^ Nuffield Department of Primary Care Health Sciences University of Oxford Oxford UK; ^2^ Institute of Applied Health Research University of Birmingham Birmingham UK

**Keywords:** financial incentive, normalisation process theory, obesity, primary care, weight management

## Abstract

In 2021 a Nationally Enhanced Service (NES) incentive for weight management in primary care was rolled out in England. This paid general practices £11.50 for every eligible referral they made to a weight management programme. We explored primary care staff's perceptions, experiences and attitudes toward the NES by conducting 37 semi‐structured interviews with General Practitioners (GPs), administrative staff and nurses preceding the introduction of the NES (May to September 2021) and 1 year later following its introduction (September to December 2022). Data were analysed using normalisation process theory. The NES for weight management solidified the position of staff already supportive of referring patients to weight management programmes. For staff less supportive of weight management services, the dissonance between the perceived lack of benefit of services and making referrals to services was reduced with referrals becoming more habitual. Facilitators to implementation included the presence of a coherent national policy; having a ‘champion’ explain key aspects; and a financial incentive if framed as benefiting the practice at large. Barriers included a perception that primary care has been shouldered with a complex and difficult health crisis; a worry over workload burdens; and inefficient and unclear referral systems. The implementation of the NES was broadly welcomed and accepted by primary care staff. Interviewees expressed concerns around the acceptance of weight management policies in primary care, the provision of training to raise the topic of weight and whether the responsibility of weight management fell with primary care, public health or with the patient.

## 1 INTRODUCTION

The prevalence of obesity is increasing worldwide, and this creates substantial early onset morbidity and consequent healthcare expenditure.^1^ In 2021, an estimated 25.9% of adults in England were living with obesity^2^ and the estimated annual cost of obesity in England in 2020 (measured in 2021 prices) was £50 billion.^3^ As a result, national public health and clinical guidelines in many countries suggest clinicians should refer people living with obesity to weight management services as part of that response.^4^ However, data from the United Kingdom, where this recommendation is part of national guidelines,^5^ suggest that referrals in this area are infrequent.^6^ Clinicians cite a lack of confidence, scepticism about patients' ability to change and sensitivity around overweight and obesity for their reluctance in offering weight management advice and support.^7^, ^8^, ^9^, ^10^ Beyond that, we identified factors related to weight stigma and perceptions of providing care which go beyond reported barriers and may explain their reluctance in a deeper way.^11^

In general, governments have typically addressed the clinical underactivity in providing preventative care through pay for quality schemes.^12^ Systematic reviews of monetary incentives have shown that they change clinicians' behaviour^13^ and are acceptable^14^ but their impact on patient care overall appears modest.^15^ In 2020, the UK government obesity strategy included guidance for primary care services to address obesity and support referral to weight management programmes. This was based on evidence that brief opportunistic interventions for people with obesity are acceptable and evidence that simple inexpensive weight management programmes are effective and cost‐saving for the NHS.^16^, ^17^ In 2021, a Nationally Enhanced Service (NES) incentive scheme was rolled out in England, in which, weight management services were made available to people with obesity who had diabetes or hypertension. This added to the existing provision of a national diabetes prevention service, a national diabetes remission service for people with overweight and obesity and newly diagnosed Type 2 diabetes and reasonably widespread but not universal provision of weight management support for anyone with obesity made available through public health systems. Additionally, GP practices opting into NES received £11.50 incentive for every eligible referral made to a weight management programme for any of these services. Practices are run as private businesses, so these earnings could directly benefit the take‐home pay of GPs, many of whom are partners in the business.

This incentive strategy may address the reasons why overweight and obesity are typically not addressed in primary. First, to maximise earnings, practices need to organise systems of care to make referrals and knowledge of and creation of new opportunities may reduce ‘friction’ in the system for clinicians; this could mean referrals are more likely because they are easier to implement, and the system is more well known. Second, the incentive may mean clinicians have conversations with patients about their weight more frequently, and this ‘reinforced’ practice is associated with greater ease among clinicians in engaging in this behaviour.^18^ Third, enforced practice could change hearts and minds. Cognitive dissonance can occur when a person acts against their values. Clinicians typically feel weight management is of low value,^7^ but typically see themselves as acting in the patient's best interest and that interest includes not discussing obesity.^11^ Acting for profit in this way creates cognitive dissonance, which could be resolved by re‐examining their values ascribed to supporting patients to manage their weight. In these ways, a simple incentive could change clinicians' hearts and minds. In this study, we examine how members of the primary care team thought and felt about weight management before and after the introduction of the NES for weight management in England and whether the incentive scheme led to changes that could outlast any withdrawal of the incentive.

## 2 METHOD

### 2.1 Recruitment of participants

We recruited general practice staff (general practitioners, nurses, other clinicians and administrators) over 18 years of age and currently working in general practice or out‐of‐hours primary care in England for at least a year. We sent flyers to all Clinical Research Networks (CRN) in England asking individuals meeting the eligibility criteria to contact us and let us know whether they were pro, neutral, or against obesity treatment in primary care. Sampling was iterative and responsive to our reflections developed from the data. We also specifically sought participants who were sceptical about weight management by advertising for them because initial recruitment yielded mainly enthusiasts. As we observed patterns and similarities in views on weight loss provision, we sampled further based on those observations to ensure maximum variation was achieved. Recruitment ceased when the sample reached information power^19^—that the data collected holds sufficient information to address the research question.

### 2.2 Ethical considerations

All participants gave informed consent prior to participation and re‐confirmed their consent post‐interview. Study information was available via participant information sheets. Participants were not informed that this was an evaluation of the NES incentive policy as we sought to gauge their experiences and beliefs about weight management in primary care before and after the policy was implemented without prompting them to assess the degree to which the policy had enacted change within the practice. Participants were debriefed after the second interview and provided the opportunity to withdraw consent. Ethical approval was granted from the Health Research Authority (reference number: 292432).

### 2.3 Data collection

One qualitative researcher, who was unknown to the participants beforehand, conducted and audio‐recorded interviews using a semi‐structured interview guide based on normalisation process theory (NPT), stakeholder input and co‐created by members of the public who have experience as patients in the NHS, living with obesity (Table 1). Participants were interviewed twice, once before and once about 12 months after the policy was introduced. All interviews were conducted by telephone. Two interview guides were used for the initial and for 1‐year interviews (see Supplements 1a and 1b). The interview guides were informed by NPT,^20^ feedback from NHS England, and PPI input to focus on thoughts and feelings about weight management and the key implementation outcomes we sought to assess. Participants were reimbursed at rates set by the CRN.

**TABLE 1:** NPT's four constructs.

1. *Coherence*, sense‐making that facilitates or inhibits acceptance of an intervention. Implementation is supported if participants can understand the intervention.
2. *Cognitive participation*, processes supporting or inhibiting the legitimisation of an intervention. Implementation is enhanced if there is support for the intervention.
3. *Collection action*, work involved in enacting an intervention. Implementation is supported with effort given by participants to integrate the intervention.
4. *Reflexive* monitoring, how the intervention is comprehended in context. Implementation is supported if participants can appraise how interventions affect practice.

Abbreviation: NPT, normalisation process theory.

### 2.4 Data analysis

All participants were assigned an anonymous participant ID. Audio‐recordings of the interviews were transcribed verbatim using a third‐party service. Both before and after NES interview data were deductively and systematically coded using a trajectory analysis^21^ within a framework method^22^ to understand how processes and experiences change over time in the same group of participants. This resulted in two framework matrices for interviews conducted at Time 1 and Time 2. We then conducted inductive axial coding in order to capture the meaning for implementation outcomes across both time points. Following coding, we analysed both interview sets using the constant comparative method^23^ to interrogate (in)consistencies in views across both interviews. The approach involves comparing individual transcripts (e.g., the same interviewee), transcripts within the same cohort (e.g., administrative staff) and transcripts across the dataset to identify differences and similarities. Contemporaneously, NPT was used as a framework for the analysis. This allowed for contrasting and complementary perspectives to be explored while evaluating them in terms of the implementation outcomes. Data retrieval and analysis were facilitated by NVivo 1.6. Reporting follows the Consolidated Criteria for Reporting Qualitative Studies.^24^

### 2.5 Normalisation process theory

Evaluating the implementation of interventions such as offering a financial incentive to encourage weight management referrals can inform the development of policies and explain whether and how implementation was successful or not. Using a theory such as NPT supports the evaluation of how interventions and practices become routine (normalised) in healthcare.^25^ Qualitative studies using NPT have tested, assessed and stabilised its core constructs—interrogating key processes underpinning implementation in very different settings, such as in community‐based healthcare, maternity care and in multi‐national primary care.^26^, ^27^, ^28^ NPT is used to explain facilitators and barriers to implementation^29^ by drawing on the generative process underpinning implementation. It concerns the individual and collective work of embedding interventions into routine practice; the work of enacting interventions in practice; and the investment and support of individuals to take forward an intervention. These concerns are represented by four constructs.

## 3 RESULTS

In total, 37 people contributed to our study: 16 GPs, 6 nurses, 5 administrators, 3 receptionists, 3 pharmacists, 2 social prescribers, 1 practice manager and 1 health coach. Thirty‐seven were initially interviewed and 36 after 1 year (1 nurse was unavailable for interview following NES' introduction). The Index of Multiple Deprivation (IMD) decile average for participants' practices was 5.18 ± 2.55 (mean ± standard deviation). IMD is calculated via postcode data with 1 = practices in most deprived areas and 10 = practices in least deprived areas.^30^ Our findings are organised around NPT's four constructs and sub‐themes developed based on these constructs and on the data (Table 2); views from before and after the NES are presented within each sub‐theme. Differences and similarities in how participants appraised the introduction of the NES intervention are discussed within each construct. We refer to individuals using anonymised identifiers. Quotes are used to illustrate findings.

**TABLE 2:** Summary of constructs and sub‐themes.

Coherence	Responsibility of raising weight
	Perceived increase in service provision
	Understanding NES as a coherent weight management policy
Cognitive participation	GP practices shouldering weight management
	Embracing a flexible approach to weight management
	Perceived value of clinicians' time in contrast to perceived value of interventions
	Perceived value for money as a barrier to support
Collective action	Perceived time to raise weight
	Quick delivery hampered by inefficient systems
	Training and support
Reflexive monitoring	Impact of a NES for weight management
	Raising weight is the right thing to do
	QOF

Abbreviations: NES, Nationally Enhanced Service; QOF, Quality and Outcomes Framework.

### 3.1 Coherence: Understanding and accepting a NES for weight management

*Coherence* represents how participants understand an intervention's purpose, how they understand that intervention to be different from previous ways of working and the degree to which participants accept the intervention (Table 3).

**TABLE 3:** Illustrative quotes for coherence.

Quote number	Participant ID	Quote
**Subtheme: Accepting responsibility to raise the topic of weight with patients**		
1	Participant 17, GP	‘I think we've all got a responsibility towards it […] we all have to take our share in responsibility here’
2	Participant 19, GP	‘Managing and tackling obesity should be, for example, the same as safeguarding. It is everybody's responsibility to do what they can within their remit’
3	Participant 8, Health Coach	‘Oh my gosh from when I spoke to you last time 100% more [referrals]’
4	Participant 26, Nurse	‘And now … since I spoke to you last, actually we have more options to help patients and support patients […] which is really very good’
5	Participant 5, Nurse	‘I actually think doing this [NES] has made it easier for me to sort of say to them [patients] you know you are a bit overweight or you know and it could be an issue with your health in the long term, have you ever you know, tried anything to lose some weight’
**Subtheme: Perceived increase in service provision**		
6	Participant 10, GP	‘We have a tier 3 weight management service, which isn't in [local area], it's in [not local area], which is not so bad now that everything is virtual but it can be quite prohibitive because some of our patients who can't afford to travel, don't have access to cars, that kind of thing. So, we do have that, it is possible, but it's not always easy’
7	Participant 16, GP	‘Digital weight management is not going to be the way they're going to change their lifestyle. It seems utterly ridiculous to me and a complete waste of money’
8	Participant 15, Nurse	‘We do have access to a lot of other ones [weight management programmes], so I wouldn't necessarily go for Digital Weight Management’
**Subtheme: Understanding the NES as a coherent weight management policy**		
9	Participant 12, GP	‘I don't think it [obesity] should be an NHS burden, actually I think it's, in my opinion, obesity has now become a public health crisis’
10	Participant 19, GP	‘I think the NHS and the Government should be investing more money in it [obesity] because I'm a very firm believed of an ounce of prevention is worth a pound of cure’
11	Participant 5, Nurse	‘The Enhanced Services, putting the right letters and numbers onto somebody's record, people learn how to play the game’
12	Participant 28, Nurse	‘It was fine because it was trying to bring everyone up to a certain standard. If you worked in a decent service, you would achieve those things. It was fine. I think now it's probably served its purpose and it's a tick box exercise’

#### 3.1.1 Accepting responsibility to raise the topic of weight with patients

Many participants described that it was very difficult to raise the topic, with many staff reporting that they avoided having weight‐related conversations. They also believed there was a shared responsibility to motivate weight loss. Single encounters/mention of weight management were not seen to be advantageous.

**Table:** 

‘I think we've all got a responsibility towards it […] we all have to take our share in responsibility here’ (Participant 17, GP)
‘Managing and tackling obesity should be, for example, the same as safeguarding. It is everybody's responsibility to do what they can within their remit’ (Participant 19, GP)

The view of sharing responsibility did not persist following NES's introduction; instead, raising the topic of weight was described as an individual act for the clinician. NES appeared to motivate clinicians to talk about weight, and this was strongly supported by non‐GP participants who were more optimistic about raising weight because they had a reason to do so.

**Table:** 

‘Oh my gosh from when I spoke to you last time 100% more [referrals]’ (Participant 8, Health Coach)
‘And now … since I spoke to you last, actually we have more options to help patients and support patients […] which is really very good’ (Participant 26, Nurse)
‘I actually think doing this [NES] has made it easier for me to sort of say to them [patients] you know you are a bit overweight or you know and it could be an issue with your health in the long term, have you ever you know, tried anything to lose some weight’ (Participant 5, Nurse)

#### 3.1.2 Perceived increase in service provision

Participants lamented the lack of resources and dwindling provision of weight management services prior to NES's introduction. They expressed this as a significant barrier to them making referrals. For many patients, weight management programmes were unavailable or inaccessible.

**Table:** 

‘We have a tier 3 weight management service, which isn't in [local area], it's in [not local area], which is not so bad now that everything is virtual but it can be quite prohibitive because some of our patients who can't afford to travel, don't have access to cars, that kind of thing. So, we do have that, it is possible, but it's not always easy’ (Participant 10, GP)

The addition of weight management programmes in NES appeared to change some minds, but this varied, with several participants describing the programmes as not worthwhile ‘lots of them have very muted feedback’ (Participant 16, GP). Most participants were particularly disparaging of the digital weight management programme because of a perceived lack of accessibility for and interest from patients.

**Table:** 

‘Digital weight management is not going to be the way they're going to change their lifestyle. It seems utterly ridiculous to me and a complete waste of money’ (Participant 16, GP)
‘We do have access to a lot of other ones [weight management programmes], so I wouldn't necessarily go for Digital Weight Management’ (Participant 15, Nurse)

#### 3.1.3 Understanding the NES as a coherent weight management policy

Participants strongly desired a government‐led policy that emphasised the importance of addressing rising rates of obesity and overweight.

**Table:** 

‘I don't think it [obesity] should be an NHS burden, actually I think it's, in my opinion, obesity has now become a public health crisis’ (Participant 12, GP)
‘I think the NHS and the Government should be investing more money in it [obesity] because I'm a very firm believed of an ounce of prevention is worth a pound of cure’ (Participant 19, GP)

Offering a financial incentive as a policy was, however, viewed pessimistically. The majority of staff made sense of the incentive as a short‐term solution to raising the performance of other practices, but more established practices could work the system to their advantage.

**Table:** 

‘The Enhanced Services, putting the right letters and numbers onto somebody's record, people learn how to play the game’ (Participant 5, Nurse)
‘It was fine because it was trying to bring everyone up to a certain standard. If you worked in a decent service, you would achieve those things. It was fine. I think now it's probably served its purpose and it's a tick box exercise’ (Participant 28, Nurse)

Views and attitudes towards weight management changed very little following the NES introduction. The majority of participants implementing NES in their practice viewed weight management referrals as not a core part of their practice ‘it just seems crazy, just an extra thing to remember and find and search for and code and everything else’ (Participant 5, Nurse).

### 3.2 Cognitive participation: Supporting the implementation of a NES for weight management

*Cognitive participation* represents how participants supported the implementation of a NES for weight management. Whether/how they view the intervention as part of their role, and in general, how the implementation was supported within their practice (Table 4).

**TABLE 4:** Illustrative quotes for cognitive participation.

**Subtheme: GP practices shouldering weight management**		
13	Participant 25, GP	‘I think we definitely do see it as part of our role. I think, often one of the problems in initiating those conversations is that there are so many other elements going on in a consultation, that … that element often gets squeezed out in favour of other things that are felt to be more of a priority. But actually, um, when we have time to do it, I think it definitely is part of our world’
14	Participant 26, Nurse	‘I think it's quite important, really because if you ignore it, as a health professional, you … you're almost condoning being overweight’
15	Participant 4, GP	‘I think this is the kind of thing that I think would—should be a public health thing, it's a public health problem and I think the solutions are probably best delivered through the community sort of not tied to GP practices, I think that would absolutely be a better idea’
16	Participant 10, GP	‘The problem is I think there are so many targets to hit at the moment with QOF and everything. I think everyone in general practice just feels a little bit targeted out and trying to do that on top of the day job is literally impossible’
**Subtheme: Embracing a flexible approach to weight management**		
17	Participant 19, GP	‘With obesity being such a complex and emotive topic, I think if you try to approach it in such a formulaic way, you would scare off a lot of patients who perhaps might have been open to doing things’
18	Participant 12, GP	‘I can probably be little more proactive in regards to it [obesity]. I think if there was more cheaper or accessible resources to be able to offer patients, I think that will be a good option’
19	Participant 31, GP	‘We prefer people to self‐refer to us than for you to refer because they're more likely to engage with the service’
20	Participant 31, GP	‘If someone signs up for something themselves and makes the effort they'll be more likely to go through with it. […] I think we have to believe that people have autonomy and we should respect that and allow them to make the decisions for their own health’
21	Participant 27, Admin	‘It would make patients more, a little bit more motivated, thinking they've got things readily available rather than just having to wait for a GP appointment’
**Subtheme: Perceived valued of clinicians' time in contrast to perceived value of interventions**		
22	Participant 7, Receptionist	‘The weight kind of thing I think it definitely has got to come from a nurse’
23	Participant 13, GP	‘Nurses tend to have less formal relationships with the patients. And so I suspect the nurses are probably the absolute best place’
24	Participant 4, GP	‘I think most GPs probably feel that there's more than enough to get on with anyway, so any time there's something new that comes along it's just one added thing’
**Subtheme: Perceived value for money as a barrier to support for implementation**		
25	Participant 34, GP	‘Has anybody looked so they know the answer to this as to whether primary care interventions are more cost effective than spending the money in public health’
26	Participant 5, Nurse	‘We're not just referring so we can get X‐amount of money, you want that patient to cost the NHS less, you want them to be in control of their own health’
27	Participant 36, Health Coach coordinator	‘£11.50 is it worth my while? Which is a terrible thing to say’
28	Participant 6, GP	‘I'm not a partner so it doesn't have like monetary, you know, benefits for me’
29	Participant 31, GP	‘For me the extra work for a thousand pounds isn't worth it. I'm now salaried which means I'm gonna see no benefit’
30	Participant 19, GP	‘If we do end up making money from it, then it gives us the ability to expand other things within the practice that help our patients. So it's kind of a win‐win’
31	Participant 12, GP	‘Even if it's going to be something nominal, like £11.50, like I said, a reimbursement, it makes a big difference towards whether the practice will engage or not […] at least that will cover our admin costs […] it makes a big difference to them’
32	Participant 25, GP	‘The Enhanced Service, I think part of that depends on the financial remuneration that's associated with it because of course that will all go back into the pot which then helps to fund the practice’

Abbreviation: QOF, Quality and Outcomes Framework.

#### 3.2.1 GP practices shouldering weight management

Prior to the introduction of the NES, most participants viewed their role in weight management as ‘initiating conversations’ with patients. Some offered caveats that those conversations were to signpost people to more equipped professionals like dieticians (see *accepting responsibility to raise weight*).

**Table:** 

‘I think we definitely do see it as part of our role. I think, often one of the problems in initiating those conversations is that there are so many other elements going on in a consultation, that…that element often gets squeezed out in favour of other things that are felt to be more of a priority. But actually, um, when we have time to do it, I think it definitely is part of our world’ (Participant 25, GP)
‘I think it’s quite important, really because if you ignore it, as a health professional, you…you're almost condoning being overweight.’ (Participant 26, Nurse)

Participants expressed different views following NES' introduction. While some continued to hold the view that their role was in initiating weight conversations, most GP participants suggested that it is not within the remit of GP practices but for public health generally to address weight with people.

**Table:** 

‘I think this is the kind of thing that I think would‐‐ should be a public health thing, it’s a public health problem and I think the solutions are probably best delivered through the community sort of not tied to GP practices, I think that would absolutely be a better idea’ (Participant 4, GP)

The change in how participants viewed their role in raising the topic of weight with patients was not attributed to a lack of willingness to raise weight, but instead to a resistance towards policies that shouldered the work onto GP practices.

**Table:** 

‘The problem is I think there are so many targets to hit at the moment with QOF and everything. I think everyone in general practice just feels a little bit targeted out and trying to do that on top of the day job is literally impossible’ (Participant 10, GP)

#### 3.2.2 Embracing a flexible approach to weight management

Prior to NES' introduction GP participants reported a desire for flexibility in broaching weight management with patients. They viewed raising the topic of weight as complex and emotive and felt patients' would require bespoke support. Moreover, some participants indicated a preference for a self‐referral system for two reported reasons^1^: patients would be more willing to adhere to programmes if they are already interested; and^2^ patients are unlikely to book a GP appointment to discuss weight management support.

**Table:** 

‘With obesity being such a complex and emotive topic, I think if you try to approach it in such a formulaic way, you would scare off a lot of patients who perhaps might have been open to doing things’ (Participant 19, GP)
‘I can probably be little more proactive in regards to it [obesity]. I think if there was more cheaper or accessible resources to be able to offer patients, I think that will be a good option’ (Participant 12, GP)
‘we prefer people to self‐refer to us than for you to refer because they’re more likely to engage with the service’ (Participant 31, GP)

Participants continued to believe that it would be better for patients to refer themselves to weight management programmes without GP involvement. They did not view using consultation time to undertake this work as a worthwhile use of time, particularly because they thought it would be a lengthy process.

**Table:** 

‘if someone signs up for something themselves and makes the effort they’ll be more likely to go through with it. [..] I think we have to believe that people have autonomy and we should respect that and allow them to make the decisions for their own health’ (Participant 31, GP)
‘It would make patients more, a little bit more motivated, thinking they’ve got things readily available rather than just having to wait for a GP appointment’ (Participant 27, Admin)

#### 3.2.3 Perceived value of clinicians' time in contrast to perceived value of interventions

Some participants before NES' introduction suggested it was not in a clinical remit to raise weight with patients ‘I don't think it [raising weight] needs to a clinical role’ (Participant 29, Pharmacist). However, many had conflicting views about this, that they perceived their nursing team/health coaches as best placed to raise weight with patients though some viewed GPs advice as having more effect: ‘you might get a bit more buy‐in if you have a doctor saying it’ (Participant 6, GP).

**Table:** 

‘the weight kind of thing I think it definitely has got to come from a nurse’ (Participant 7, Receptionist)
‘Nurses tend to have less formal relationships with the patients. And so I suspect the nurses are probably the absolute best place’ (Participant 13, GP)

Clinicians' views on the worth of discussing obesity with their patients following NES introduction did not change. They valued their time highly, and this was a barrier to acting on the incentive payment available in the enhanced service. They believed the solution was elsewhere and not in asking clinicians to do more.

**Table:** 

‘I think most GPs probably feel that there’s more than enough to get on with anyway, so any time there’s something new that comes along it’s just one added thing’ (Participant 4, GP)

#### 3.2.4 Perceived value for money as a barrier to support for implementation

Prior to the introduction of the NES, financial support for practices for weight management referrals was rarely mentioned. One participant mentioned that including weight loss support as part of the Quality and Outcomes Framework (QOF)* for obesity (an incentive scheme that asks clinicians to weigh people on the obesity register once a year) would be advantageous. However, most participants supported more investment in population‐level preventative public health policies.

**Table:** 

‘has anybody looked so they know the answer to this as to whether primary care interventions are more cost effective than spending the money in public health’ (Participant 34, GP)

Clinicians were sceptical that weight loss interventions were cost‐effective for the NHS and this deterred them from referring patients. One participant said, ‘I suppose if they haven't even tried simple strategies themselves, then that would be the first step, really, before you necessarily involve the expense and professionals’ (Participant 17, GP).

Similar views were offered following the introduction of the NES. Several clinicians evaluated the NES as a ‘brazen’ payment which did not motivate them because it was not about the wellbeing of the patient.

**Table:** 

‘we’re not just referring so we can get X‐amount of money, you want that patient to cost the NHS less, you want them to be in control of their own health’ (Participant 5, Nurse)

Many participants did not perceive the money as directly benefitting them and were therefore not motivated to invest energy in delivering the enhanced service.

**Table:** 

‘£11.50 is it worth my while? Which is a terrible thing to say’ (Participant 36, Health Coach Coordinator)
‘I’m not a partner so it doesn’t have like monetary, you know, benefits for me’ (Participant 6, GP)
‘For me the extra work for a thousand pounds isn’t worth it. I’m now salaried which means I’m gonna see no benefit’ (Participant 31, GP)

Participants understanding that the incentive benefitted their practice were somewhat pleased and offered more support towards the NES.

**Table:** 

‘If we do end up making money from it, then it gives us the ability to expand other things within the practice that help our patients. So it’s kind of a win‐win’ (Participant 19, GP)
‘Even if it’s going to be something nominal, like £11.50, like I said, a reimbursement, it makes a big difference towards whether the practice will engage or not [..] at least that will cover our admin costs [..] it makes a big difference to them’ (Participant 12, GP)
‘The Enhanced Service, I think part of that depends on the financial remuneration that’s associated with it because of course that will all go back into the pot which then helps to fund the practice’ (Participant 25, GP)

### 3.3 Collective action: enacting and integrating a NES for weight management

*Collective action* represents the labour involved to implement the NES for weight management. It relates to whether the actions needed to enact this policy can be integrated into existing work and the provision of training and resources to support implementation (Table 5).

**TABLE 5:** Illustrative quotes for collective action.

**Subtheme: Perceived time required to raise the topic of weight**		
33	Participant 4, GP	‘I think if we're talking about genuinely supporting the process of losing weight then I think it would be the nursing team, just because I guess, I think a one‐off consultation is very unlikely to make a difference’
34	Participant 31, GP	‘So if a health care assistant takes someone's blood then they should be able to sow seeds and talk about weight management’
35	Participant 24, Pharmacist	‘I think it's got to be a team approach. […] It's … it's very difficult for GPs to have those conversations when they're so time limited’
36	Participant 13, GP	‘The detail, the time is in that discussion and the emotions around that discussion and assessing people's readiness to change um … and that takes a lot of time but we should be doing that anyway um … so in that sense it's not sort of extra but sort of get all the boxes ticked for the enhanced service, it is actually quite a lot for not a very good payment’
**Subtheme: Quick delivery hampered by systems**		
37	Participant 6, GP	‘It's a very quick thing then I might mention at the end kind of, oh I also notice that—and I kind of phrase it in a way that you'd be eligible for weight management services’
38	Participant 10, GP	‘By the time you've filled in all the forms which aren't particularly long but just EMIS is so slow if any of it's being tagged on the end of an already running late 10‐minute consultation, it's just another three minutes that you don't have … it feels very tick box at the moment’
39	Participant 11, GP	‘It's a bit clunky to refer patients. They keep changing their form. And if you've done the form that was the form two months ago, that's no good. And you have to redo it again with another one’
**Subtheme: Training and support**		
40	Participant 5, Nurse	‘We need more training and maybe then we can actually offer them [patients] something that is worthwhile rather than paying it lip service’
41	Participant 19, GP	‘The kind of people that I work with, they've very proactive and very much wanting to help their patients. So it's less of a “Oh, yes. But I'm now going to start doing this because I'm getting paid for it’ and more of a ‘oh, there's a now a service”'
42	Participant 6, GP	‘The partners are very on it though with things like incentives and they do very well at honing down to us that this is beneficial for the practice and if it's beneficial for the practice eventually it will be beneficial for us’
43	Participant 32, Admin	‘One of our um … senior partners, he's obviously … he's really on the ball with um … the QOF situation […] he has sent us emails and actually we had sort of a team event a couple of weeks ago and he sort of did talk and explained about QOF because even the clinicians, a lot of them didn't know and he explained how the money comes in and you know how we're funded’

Abbreviation: QOF, Quality and Outcomes Framework.

#### 3.3.1 Perceived time required to raise the topic of weight

Participants perceived that an optimal weight management intervention would be lengthy and require staff to be available to provide specific and continued support. Notably, the ‘team approach’ excluded GPs on the basis of the time required to raise weight.

**Table:** 

‘I think if we’re talking about genuinely supporting the process of losing weight then I think it would be the nursing team, just because I guess, I think a one‐off consultation is very unlikely to make a difference’ (Participant 4, GP)
‘So if a health care assistant takes someone’s blood then they should be able to sow seeds and talk about weight management’ (Participant 31, GP)
‘I think it’s got to be a team approach. [..] It’s… it’s very difficult for GPs to have those conversations when they’re so time limited’ (Participant 24, Pharmacist)

Perceptions of the time needed to raise weight did not change following the introduction of the enhanced service. GP's expressed worries that weight related conversations required detailed discussions, emotional support and a need for them to assess a patient's ‘readiness to change’.

**Table:** 

‘the detail, the time is in that discussion and the emotions around that discussion and assessing people’s readiness to change um… and that takes a lot of time but we should be doing that anyway um… so in that sense it’s not sort of extra but sort of get all the boxes ticked for the enhanced service, it is actually quite a lot for not a very good payment’ (Participant 13, GP)

#### 3.3.2 Quick delivery hampered by systems

Several participants reported having a preexisting system for prompting weight management referrals but the impact of these were unclear. Some required the clinician to ‘click the right buttons and it will flag up that they've got obesity’ (Participant 11, GP), or ‘often have little pop‐up flags as well on the software that do alert us to say, you know, “BMI … obesity check due” (Participant 17, GP). While this raised awareness of weight management generally, the perception that weight management discussions needed to take more time than was available persisted: “we still have 10‐minute appointments and it's not really a 10‐minute conversation”’ (Participant 10, GP).

Following the introduction of NES, clinicians were less likely to say that discussing obesity took a long time. The majority of participants commented on the relative ease and speed of raising weight and preparing a referral.

**Table:** 

‘It’s a very quick thing then I might mention at the end kind of, oh I also notice that‐‐ and I kind of phrase it in a way that you’d be eligible for weight management services’ (Participant 6, GP)

Despite this perception changing, clinicians remained reluctant to make a referral because of time constraints. This was no longer caused by the time taken in consultation but because they perceived that the technology and bureaucracy involved in making a referral were onerous and lengthened the consultation beyond 10 min.

**Table:** 

‘By the time you’ve filled in all the forms which aren’t particularly long but just EMIS is so slow if any of it’s being tagged on the end of an already running late 10‐minute consultation, it’s just another three minutes that you don’t have… it feels very tick box at the moment’ (Participant 10, GP)
‘It’s a bit clunky to refer patients. They keep changing their form. And if you’ve done the form that was the form two months ago, that’s no good. And you have to redo it again with another one’ (Participant 11, GP)

#### 3.3.3 Training and support

Before the NES, clinicians commonly reported that they did not have sufficient training and support to raise weight. Many believed they were paying ‘lip service’ to weight management and not meaningfully supporting patients to manage their weight.

**Table:** 

‘We need more training and maybe then we can actually offer them [patients] something that is worthwhile rather than paying it lip service’ (Participant 5, Nurse)

Training and resources to support implementing the NES for weight management varied between practices. Many received no training or support, some had online training modules, some received emails ‘so we're just reading up and people sort of taking what they will from that’ (Participant 4, GP), some had a designated lead who trained other staff. Participants working in practices with a champion who supported weight management were positive about the value of weight management. These participants viewed the additional support for weight management as more important than the financial incentive.

**Table:** 

‘The kind of people that I work with, they’ve very proactive and very much wanting to help their patients. So it’s less of a ‘Oh, yes. But I’m now going to start doing this because I’m getting paid for it’ and more of a ‘oh, there’s a now a service'’ (Participant 19, GP)

Endorsement of the incentive from partners enhanced support for its implementation in practices. Buy‐in at a senior level supported participants' understanding of the incentive as benefiting the practice as a whole, and these participants reported commitment to referring patients to weight management programmes.

**Table:** 

‘The partners are very on it though with things like incentives and they do very well at honing down to us that this is beneficial for the practice and if it’s beneficial for the practice eventually it will be beneficial for us’ (Participant 6, GP)
‘One of our um… senior partners, he’s obviously… he’s really on the ball with um… the QOF situation [..] he has sent us emails and actually we had sort of a team event a couple of weeks ago and he sort of did talk and explained about QOF because even the clinicians, a lot of them didn’t know and he explained how the money comes in and you know how we’re funded’ (Participant 32, Admin)

Participants who supported and were committed to referring patients to weight management programmes had complementary views linking NES engagement with benefits to their whole practice, and similarly, understood the benefits of weight management programmes for patients.

### 3.4 Reflexive monitoring: Appraising a NES for weight management

*Reflexive monitoring* represents how participants appraised the NES for weight management. This includes being cognisant of the impact of the policy, whether they viewed the policy as worthwhile, and their views on the evolution of the NES (Table 6).

**TABLE 6:** Illustrative quotes for reflexive monitoring.

**Subtheme: Impact of a NES for weight management**		
44	Participant 19, GP	‘It would be interesting to see the statistics on how well it is actually working. So, what number of patients actually use the programme? Which ones continue? Do these lose weight?’
45	Participant 4, GP	‘I think most of us now are very aware that brief interventions have relatively little impact on you know, in supporting significant weight loss’
46	Participant 33, Admin	‘I just feel we're going through the motions. We're doing our part of the referral, but they're [patients] not sort of seeing it through’
47	Participant 6, GP	‘So, I have noticed I've been making more referrals, now whether that's because I've been more proactive or whether I feel that patients, there are more patients that are more accepting of this, I'm not sure. Maybe it's a combination’
48	Participant 11, GP	‘Following the introduction of the enhanced service, there's more discussion within the practice about more proactive identification of such patients’
49	Participant 13, GP	‘I don't know if I'm bringing it up more but certainly it's making it easier because there's an easier solution for them of it so with it being a local one, it feels relevant to them and feels easy for them to do it’
50	Participant 17, GP	‘So, I think enhanced services can help … help prioritise relevant health professionals and draw attention as well to clinicians and the administrative staff and managers, to, you know, in a busy practice with many demands. These can sort of highlight them and make them higher in the priority list, really’
51	Participant 16, GP	‘Kind of extra push to get that done. I mean, obesity recording is one of the core things, the enhanced service bit, I've got to say hasn't been that help useful. I've referred … There's a lot of financial incentive to do it. I don't think it's very useful for patients’
**Subtheme: Raising the topic of weight is the right thing to do**		
52	Participant 30, GP	‘If I am a GP and I'm doing my job properly I should automatically be doing the right thing because it's the right thing to do. I mean if someone's diabetic with angina and they smoke, my telling them to stop smoking shouldn't be dependent on being paid to refer to a service’
53	Participant 31, GP	‘Enhanced Service comes along which is worth one or thousand pounds for a year then I would think there's gonna be lots of easier ways to make one or two thousand pounds […] And the Enhanced Service there's lots of bureaucracy attached. And then it comes back to why get someone to do something as an Enhanced Service that they should be doing because it's the right thing to do’
54	Participant 5, Nurse	‘I hate saying it but you want it [weight management] to be more result driven but not for us for the patient because we're not just referring so we can get x‐amount of money, you want that patient to cost the NHS less, you want them to be more in control of their own health and wellbeing um … yes, so yes and Enhanced Service is good but it has to be for the right reasons’
**Subtheme: QOF**		
55	Participant 11, GP	‘QOF feels like core, and enhanced services feel like extra. And always the first priority in terms of these sorts of indicators and performance management and so on … It's always … QOF always comes first’
56	Participant 10, GP	‘I think I have to kind of pick and choose a little bit which ones you're going to do um … I think also the other thing is because QOF's been there, we sort of had the systems sort of built in, so you know you have your chronic disease reviews built in and we know what we're doing with those, everybody does that but with the Enhanced Services it's just impossible to keep up with everything’
57	Participant 4, GP	‘If it was more integrated into QOF and something more regulated it would probably get a bit more attention but I think sometimes Enhanced Services kind of feel like an extra bit of work on top of everything else so I think they get a bit of attention now and then but I don't think it's very systematic’
58	Participant 4, GP	‘I think Enhanced Services, I don't know, maybe it's partly the name, maybe it's the way they're implemented, but they sometimes feel like an optional extra so I think because, you know, for whatever reason we're probably being a little bit—we're exercising more discretion in terms of who we offer it to’
59	Participant 30, GP	‘The enhanced services are … They are important, but they're kind of not core. They're outside the core. I guess if you think an extra bonus money when we need it. But you can kind of pick and choose what, you can do. You can't pick and choose your QOFs’
60	Participant 31, GP	‘If it's in QOF we'll fail to meet the targets. Cause if the target is the person will have lost 1% of their body weight and they fail, a lot of GPs would be failing QOF’
61	Participant 10, GP	‘It makes my head want to explode sometimes with all of the different things that we've got to do. I think someone said the other day we have 72 targets in general practice’

Abbreviation: QOF, Quality and Outcomes Framework.

#### 3.4.1 Impact of a NES for weight management

Most participants appraised weight management interventions negatively prior to the NES. Programmes were described as not sufficiently benefiting patients. There were two common reasons offered: first, that patients needed to be prepared to change on their own without support; and second, that programme provision was inadequate and inaccessible.

**Table:** 

‘It would be interesting to see the statistics on how well it is actually working. So, what number of patients actually use the programme? Which ones continue? Do these lose weight?’ (Participant 19, GP)
‘I think most of us now are very aware that brief interventions have relatively little impact on you know, in supporting significant weight loss’ (Participant 4, GP)
‘I just feel we’re going through the motions. We’re doing our part of the referral, but they’re [patients] not sort of seeing it through’ (Participant 33, Admin)

Following the introduction of the NES, many GP participants reported being ‘proactive’ with weight management, that they were having more discussions about weight management with their patients and within their practice team. This reflected a change in views from being against weight management services to becoming more welcoming of making referrals.

**Table:** 

‘So, I have noticed I’ve been making more referrals, now whether that’s because I’ve been more proactive or whether I feel that patients, there are more patients that are more accepting of this, I’m not sure. Maybe it’s a combination’ (Participant 6, GP)
‘Following the introduction of the enhanced service, there's more discussion within the practice about more proactive identification of such patients.’ (Participant 11, GP)
‘I don’t know if I’m bringing it up more but certainly it’s making it easier because there’s an easier solution for them of it so with it being a local one, it feels relevant to them and feels easy for them to do it’ (Participant 13, GP)

The predominant appraisal of the NES was as a policy designed to raise awareness and ‘focus the minds of some practices’ (Participant 26, Nurse) to prioritise overweight and obesity within primary care.

**Table:** 

‘So, I think enhanced services can help…help prioritise relevant health professionals and draw attention as well to clinicians and the administrative staff and managers, to, you know, in a busy practice with many demands. These can sort of highlight them and make them higher in the in the priority list, really’ (Participant 17, GP)

One participant commented that their practice only opted into the scheme to prompt staff to update patients' BMI, and that they were still uncertain about its efficacy but engaged nonetheless.

**Table:** 

‘kind of extra push to get that done. I mean, obesity recording is one of the core things, the enhanced service bit, I’ve got to say hasn’t been that help useful. I’ve referred…There’s a lot of financial incentive to do it. I don’t think it’s very useful for patients’ (Participant 16, GP)

#### 3.4.2 Raising the topic of weight is the right thing to do

The majority of participants explained that supporting patients with weight management was their moral duty and they were not doing it because of a financial incentive: ‘It's not going to change my behaviour because there's funding attached to it. But I am going to do what I think is the right thing to do’ (Participant 34, GP). This view was often supported with examples of other, more advantageous, ways of bringing money into a practice.

**Table:** 

‘If I am a GP and I’m doing my job properly I should automatically be doing the right thing because it’s the right thing to do. I mean if someone’s diabetic with angina and they smoke, my telling them to stop smoking shouldn’t be dependent on being paid to refer to a service’ (Participant 30, GP)
‘Enhanced Service comes along which is worth one or thousand pounds for a year then I would think there’s gonna be lots of easier ways to make one or two thousand pounds [..] And the Enhanced Service there’s lots of bureaucracy attached. And then it comes back to why get someone to do something as an Enhanced Service that they should be doing because it’s the right thing to do’ (Participant 31, GP)

While participants expressed a desire to do the ‘right thing’ in supporting patients with weight management, the same GP also reported ‘other areas where GPs refuse to do things because there's no funding’ (Participant 31, GP). One nurse reflected this dissonance between being morally and financially motivated to raise weight with patients:

**Table:** 

‘I hate saying it but you want it [weight management] to be more result driven but not for us for the patient because we’re not just referring so we can get x‐amount of money, you want that patient to cost the NHS less, you want them to be more in control of their own health and wellbeing um… yes, so yes and Enhanced Service is good but it has to be for the right reasons.’ (Participant 5, Nurse)

#### 3.4.3 Quality and Outcomes Framework

The additional extra of the enhanced service was appraised as offering a small boost of attention in the short term but was unfeasible as a long‐term solution because of workload demands and the lack of systematicity compared with the QOF.

**Table:** 

‘QOF feels like core, and enhanced services feel like extra. And always the first priority in terms of these sorts of indicators and performance management and so on…It’s always…QOF always comes first’ (Participant 11, GP)
‘I think I have to kind of pick and choose a little bit which ones you’re going to do um… I think also the other thing is because QOF’s been there, we sort of had the systems sort of built in, so you know you have your chronic disease reviews built in and we know what we’re doing with those, everybody does that but with the Enhanced Services it’s just impossible to keep up with everything’ (Participant 10, GP)
‘if it was more integrated into QOF and something more regulated it would probably get a bit more attention but I think sometimes Enhanced Services kind of feel like an extra bit of work on top of everything else so I think they get a bit of attention now and then but I don’t think it’s very systematic’ (Participant 4, GP)

Most offered a mixed appraisal of the enhanced service as an incentive scheme. Some remarked that weight management ought to be part of the QOF because it would then offer better remuneration and not be optional, and would be more clearly an integral part of patient care.

**Table:** 

‘I think Enhanced Services, I don’t know, maybe it’s partly the name, maybe it’s the way they’re implemented, but they sometimes feel like an optional extra so I think because, you know, for whatever reason we’re probably being a little bit—we’re exercising more discretion in terms of who we offer it to’ (Participant 4, GP)
‘The enhanced services are… They are important, but they’re kind of not core. They’re outside the core. I guess if you think an extra bonus money when we need it. But you can kind of pick and choose what, you can do. You can’t pick and choose your QOFs’ (Participant 30, GP)

But this view was not held by others, explaining the difficulty of meeting targets related to weight management. They queried how targets would be measured, and they expressed apprehension towards linking targets with funding their practice.

**Table:** 

‘If it’s in QOF we’ll fail to meet the targets. Cause if the target is the person will have lost 1% of their body weight and they fail, a lot of GPs would be failing QOF’ (Participant 31, GP)
‘It makes my head want to explode sometimes with all of the different things that we’ve got to do. I think someone said the other day we have 72 targets in general practice’ (Participant 10, GP)

## 4 DISCUSSION

In this study of 37 members of primary care staff, we assessed how the National Enhanced Service for Weight Management changed their thoughts and feelings on weight management through the lens of NPT. We found uptake and agreement with the general purpose of NES hinged on senior members of staff leading implementation of the NES and that raising weight with patients became more favourably viewed and seemingly easier to do, but uncertainty about the value of weight management, the options available to support it, and the bureaucracy of the system were deterrents. The financial incentive appeared to reduce clinician dissonance between their negative attitude towards weight management and their behaviour of making referrals to weight management services, and it confirmed the position of those who were already supportive of raising the topic of weight.

### 4.1 Coherence

The NES for weight management was widely adopted and welcomed by our participants because it offered a coherent policy on weight management. In the preceding interviews, all participants described a general fear over a diminishing availability of weight management programmes alongside a desire for a coherent policy to address obesity. In the post‐NES introduction interviews, participants felt their intent to intervene on weight was undermined because services were being withdrawn, did not work, or were inaccessible, and they felt that there was no coherent policy response to obesity. The NES was viewed as a simple incentive scheme. This appears to be a common reflection of financial incentives.^31^ For weight management, GP‐participants were wary of financial incentives as motivation, though this seemed linked to a broader concern over losing autonomy^32^, ^33^ but this view was not shared by other participants. Many participants welcoming the NES did so because they had a reason for raising the topic of weight with patients and because NES had been adopted by senior members of staff. Evidence suggests having a sense of involvement can engender a greater sense of control and encourage participation.^34^, ^35^ It may be that promoting autonomy may support acceptance,^31^ but it is unclear whether efforts to promote acceptance would markedly change practice.

### 4.2 Cognitive participation

Prior to NES's introduction, participants viewed overweight and obesity as the responsibility of individuals, society, the government and not primary care echoing previous studies on GPs' views on weight management.^36^ It was perceived to be more advantageous to allow self‐referral into weight management programmes because patients would be more willing—though evidence contradicts this.^9^ This was compounded by views from across all staff that non‐GPs (nurses, health assistants, social prescribers) would be better placed to handle matters of weight with patients. Resistance was attributed towards policies placing problems at the door of primary care, and that weight management referrals are supplementary to a GP's job. Most participants, of varying roles, described GPs time as sacrosanct, with additional demands on their workload as unreasonable, exacerbated by difficult and complex referral systems.^37^ While these concerns are well documented,^38^, ^39^, ^40^ effective weight loss referrals can be very quick^41^ with good prospects for patient health outcomes.^17^ Reassuring primary care staff that raising weight can be efficient by reducing bureaucracy and the time required to make a weight management referral is likely to enhance primary care staff buy‐in.

### 4.3 Collective Action

Participants expressed a desire for a systematic approach to weight management that did not exist, such that every contact in practice could support the weight management goal,^42^ although administrative‐participants disagreed. Those who expressed this view wanted more training; other research also reports that lack of training is given as a reason not to raise weight management.^7^, ^43^, ^44^, ^45^, ^46^ In the following‐NES interviews, participants reported similar desires and those tending to be more accepting of NES featured colleagues supporting the delivery of the enhanced service. Research shows peer support of interventions is likely to increase uptake.^47^, ^48^, ^49^ The view that raising weight with patients required a drawn‐out conversation softened significantly following the NES introduction. Many reported that they could do this speedily, but complaints of inefficient systems rendering referrals as time‐consuming hampered GP‐ and nurse‐participants willingness to do so. Implementation of weight management interventions may be enhanced if primary care staff can be reassured that simple approaches requiring little training are effective,^50^ if a ‘champion’ role can be created within each practice, and if referral systems could be clearer and simple for referring clinicians.

### 4.4 Reflexive monitoring

Appraisals of a NES for weight management were generally positive among all participants, particularly those connecting the benefits to their practice and patients with the incentive. In their positive accounts of the NES, most understood it as a short‐term overweight and obesity awareness‐raising policy and not an enforced practice. The dissonance created by the NES between GP participants feeling that weight management is of low value,^7^ and responding to the incentive by making referrals appeared to be reconciled by GP participants in re‐evaluating the effect that weight management services could have on patients. All participants viewed themselves as having a moral duty to support patients, and following the introduction of the NES, it was only a few GP participants who expressed reluctance to raise weight. Incentivising referrals to become a habitual behaviour is likely to have reduced GPs dissonance.^51^, ^52^ Non‐GP participants were grateful for having a reason to raise weight, and the financial incentive added an external justification which solidified their pre‐existing support for raising weight.^51^ Participants who remained reluctant viewed weight management services as ineffective, their workload as excessive, and that the incentive was insufficient given the value of other incentives in the QOF, which GP participants perceived as mandatory. Communicating the strong body of evidence in favour of brief opportunistic weight management referrals,^17^, ^41^ and continued engagement in weight management referrals in spite of reluctance may, over time, reduce these concerns.

Our findings add to work on financial incentives in UK primary care, supporting evidence that offering financial incentives to clinicians is acceptable and changes behaviour.^6^, ^7^, ^45^ Studies have tended to focus on the limitations of financial incentives,^22^, ^44^ but our study suggests a financial incentive enhanced the accessibility of weight management services and supported raising weight to become more habitual. For participants already supportive of raising weight with patients, the NES solidified their position and lent justification for making referrals. We observed the financial incentive, while criticised for being a brazen payment burdened with bureaucracy, influenced participants' reported behaviour and reduced the dissonance between their attitudes towards weight management and their behaviour. However, most participants continued to be cautious of primary care facing policies which put the onus on primary care to address overweight and obesity.

## 5 STRENGTHS AND LIMITATIONS

A strength of this research is the varied sample in relation to the different views held and their roles within primary care, particularly sampling people with a range of views about weight management. This contributes to a fuller understanding of the implementation of incentive schemes for weight management, what effects it had upon its introduction and what may have or be hampering its implementation. This means our findings are more relevant and impactful for the design of future national policies for obesity. Another strength is the longitudinal design, which allowed us to see how a simple incentive could have effects on the system of care.

A limitation was that post‐NES interviews were conducted at roughly a 1‐year interval. It is therefore likely that views and attitudes may have changed as practices and staff became more accustomed to NES for weight management.

## 6 CONCLUSION

The implementation of a NES for weight management appears to have been broadly welcomed and accepted by primary care staff. Whilst there was some scepticism from GPs in particular, this seemed to be allayed by having a coherent policy on weight management. The financial incentive confirmed the position of participants supporting raising weight and reduced the negative feelings of those who saw little to no benefit of weight management in primary care. Implementation of future weight management policies may be improved by more straightforward referral systems, communication on the effectiveness of brief opportunistic interventions on weight management, and buy‐in from peers and senior members of staff.

## AUTHOR CONTRIBUTIONS

JBJ was responsible for conceptualisation, data analysis and writing of the original draft. AH was responsible for conceptualisation, data collection, data analysis and reviewing and editing the manuscript. RB, KJ and SAJ were responsible for funding acquisition and reviewing and editing of the manuscript. PA was responsible for conceptualisation, funding acquisition, supervision, reviewing and editing of the manuscript and writing of the original draft.

## FUNDING INFORMATION

The research was funded by the NIHR Oxford and Thames Valley Applied Research Collaboration (NIHR200173).

## CONFLICT OF INTEREST STATEMENT

PA and SAJ are investigators on two publicly funded trials in which Nestle has donated food products to support NHS treatment costs.

## Supporting information


**Data S1.** Supporting Information.

## Footnotes

* The QOF is a voluntary incentive programme for GP practices in England. Points are earned based on achievements against 5 indicators. Payments are based on points achieved relative to a practices' local area.
