# Supplementary material for: The implementation of a Nationally Enhanced Service incentive for weight management: A longitudinal qualitative study of the perceptions and experiences of UK primary care staff on weight management using normalisation process theory
Source: Clin Obes. 2025 Apr 29;15(5):e70020. doi: 10.1111/cob.70020 (PMC12401635; doi:10.1111/cob.70020)
Supplement: Supplementary file 1 — Data S1. Supporting Information. [file COB-15-e70020-s001.pdf]

Supplement 1a – Initial Interview guide

0. Before we jump in, can you please tell me about yourself and your role in ______ clinic? [All staff]
1. What is your current role in caring for patients with obesity?
    a. Prompt: What would a typical consultation look like? [Staff conducting consultations]
    b. Probe: Is initiating a conversation on weight a part of your role?
    c. Probe: Do you have a certain way of navigating a conversation on weight? [Clinicians only]
    d. Probe: Who normally brings up the topic of weight? [Staff conducting consultations]
2. In your opinion, how necessary is it for [insert professional role] to lead conversations on weight with patients with obesity? [Staff conducting consultations]
    a. Prompt: Do you think [physicians/ nurses] see it is part of their role?
    b. Probe: Is it relevant for your work? If so, why? If not, why not?
    c. Probe: Does leading conversations for the management of diabetes and/or cardiovascular disease seem more pertinent? Why or why not?
3. What advice do you typically give to your patients with obesity? [Staff conducting consultations]
    a. Probe: Do you tend to advise people or prefer to offer referral?
        i. Probe: How do you offer referral?
        ii. Probe: Is it your job to ensure the patient pursues the referral?
4. When you talk to your patients about their weight, do you think it affects your relationship with them? If so, how? If not, why not? [Staff conducting consultations]
    a. Prompt: Does it impact on the flow of the rest of the consultation?
    b. Probe: Do you have confidence that intervening on weight is appropriate and appreciated by your patients with obesity?
    c. Notes: Explore issues around trust, comfort, strategies for health promotion/support.
5. What do you think are the most effective/practical ways of helping patients with obesity manage their weight? [All staff]
    a. Probe: Do you have a way of raising the topic that you use and works for you? What do you say? [Staff conducting consultations]
6. What do you think of referring patients to a weight management service? [Staff conducting consultations]
    a. Prompt: How useful do you think they are to you/your patients?
    b. Probe: How capable do you think that your patients are in being able to lose weight through such services?

c. Probe: Based on your knowledge of the work of your clinic, how does this fit in with the flow of work your team does?

7. Outside of referrals, do you have another preferred method for weight management that you normally employ, and how does fit in with your responsibility as a [physician/nurse]? [Staff conducting consultations]
8. How will/has intervening on weight with patients with obesity become/been part of your routine work?
9. Does leading a conversation on weight affect the nature of your work? [Staff conducting consultations]
   What about making/supporting a referral to a weight management program? All-staff]
   a. Probe: How do either of these actions impact the work of your clinic collectively?
   b. Probe: Are staff on the same page? Why or why not?
10. Tell me about what your clinic does to manage, support, and provide resources for patients with obesity who are trying to lose weight? [All-staff]
    a. Prompt: How equipped is your practice to support patients with obesity to lose weight? Do you feel what you do is appropriate?
11. Who is best positioned to promote weight loss in your practice's patients, if anyone? [Staff conducting consultations]
    Is it a relevant part of your role as a _______ to support patients in their weight management treatment protocol? [All-staff]
12. What are some of the barriers to supporting your patients with obesity to lose weight? Have you found something that makes this easier for you and the patient? [All-staff]
13. Do you feel you can and should modify the way you approach weight management for your patients with obesity? Please explain. [Staff conducting consultations]
14. If you could write a guideline for weight management for people with obesity in the UK, what would be in your guideline? [Staff conducting consultations]

Supplement 1b – One year post-NES introduction interview

0. *Before we jump in, can you please remind me about yourself and your role in your surgery?* [All-staff]
1. *What is your current role in caring for patients with obesity?* [Staff conducting consultations]
   a. *Prompt: What would a typical consultation look like?*
   b. *Probe: What is the current practice of weighing patients?*
   c. *Probe: Is initiating a conversation on weight a part of your role?*
   d. *Probe: Do you have a certain way of navigating a conversation on weight?*

e. Probe: Who normally brings up the topic of weight? (patient led, or practitioner led?)

2. *In your opinion, how necessary is it for [insert professional role] to lead conversations on weight with patients with obesity?* [Staff conducting consultations]
    a. *Prompt: Do you see it is part of your professional role? How does your profession see it, do you think?*
    b. *Probe: Is it relevant for your work? If so, why? If not, why not?*
    c. *Probe: Does leading conversations for the management of diabetes and/or cardiovascular disease seem more pertinent? Why or why not?*
3. *What advice do you typically give to your patients with obesity?* [Staff conducting consultations]
    a. *Probe: Do you tend to advise people or prefer to offer referral?*
        i. *Probe: How do you offer referral?*
        ii. *Probe: Is it your job to ensure the patient pursues the referral? Why/why not?*
4. *When you talk to your patients about their weight, do you think it affects your relationship with them? If so, how? If not, why not?* [Staff conducting consultations]
    a. *Prompt: Does it impact on the flow of the rest of the consultation?*
    b. *Probe: Do you have confidence that intervening on weight is appropriate and appreciated by your patients with obesity?*
    c. *Notes: Explore issues around trust, comfort, strategies for health promotion/support.*
5. *What do you think are the most effective/practical ways of helping patients with obesity manage their weight?* [All-staff]
    a. *Probe: Do you have a way of raising the topic that you use and works for you? What do you say?* [Staff conducting consultations]

Interview 2 guide (additions):

0. Before we jump in, you mentioned in your email that your practice [IS/IS NOT] taking part in the Enhanced Service.
    a. What is your understanding of the Enhanced Service for obesity management?
        i. [*clarify if there is any misunderstanding, so we can be on same page about what we are talking about for the rest of the interview*]
    b. Can you share your knowledge of the landscape of weight management services at your disposal as a clinician/administrative assistant? Tell me about them. [*interviewer will then go on to speak to the WMS and DWMPs, and role ES plays*]

If they are participating in the ES, go through ***version A****.*

If they are not participating in the ES, go through ***version B****.*

Version A

Coherence (sense making, differentiating from other activities)

1. In your opinion, what is the value of a programme that incentivises referrals to weight management programmes in primary care? [All staff]
2. Do you know what the enhanced service is aiming to achieve?
    a. Do you think it will achieve its aims? Please explain.
    b. Do you think the Enhanced service will benefit your patients? If so, why? If not, why not? [All staff]
3. What strategies have you used previously to manage obesity in your patients living with obesity? [Staff conducting consultations]
4. How does the Enhanced Service change what you do? [All staff]
5. Do you think that it is an important part of your professional role to discuss weight and refer patients with obesity to a weight management programme? [Staff conducting consultations]
6. In your opinion, how does the Enhanced Service aims and activity fit with the overall goals and activity of your surgery? [All staff]
7. The eligibility criteria for the Enhanced Service is: patients living with obesity and the DWMP have further requirements of a diagnosis of T2DM and/or hypertension. What do you think about this?
    a. Probe: What do you think about the scope?
    b. Probe: Is it easy or difficult to keep track of which patients qualify?

Cognitive Participation (engagement, buy-in, creating a sense of legitimacy of the action)

1. How did you learn about the Enhanced Service /who told you about the new scheme? [All staff]
2. How is your surgery delivering the Enhanced Service? [All staff]
    a. *Probe on patient-engagement- use of texts?*
3. Why was it right for you to promote weight management programmes to your patients? [Staff conducting consultations]
4. What programmes are you referring patients to? [Staff conducting consultations]
    a. What makes you select one patient for online and another for in-person (i.e. one for a locally commissioned WMS vs the DWMPs)?
    b. Are you aware of the NHS Digital Weight Management Programme?
    c. What do you think about this programme?
        i. How does this programme sit alongside other weight management offers available in your local area?
        ii. What has been the reaction of your colleagues to the NHS Digital Weight Management Programme?

iii. What has been the reaction of your patients to the NHS Digital Weight Management Programme?
iv. What has been your experience with offering a digital service?

5. The DWMP is linking the public and private sector by commissioning private weight management programmes. What do you think about this? [All staff]
6. How committed did you feel to making a referral? [All staff]
    a. Many practitioners refer to time constraints for prevention. How do you now make the time for a referral?
    b. Is there support for the patient to pursue the referral?
    c. What is the follow-up process like?
7. Was someone driving this forward or encouraging involvement in the Enhanced Service in your clinic? [All staff]
    a. Who was this?
    b. Why do you think they were motivated to promote the scheme?

Collective Action (actions and interactions required to use the intervention)

1. Does the Enhanced Service fit easily with your daily work practices or did you have to change your usual way of consulting with patients? [All staff]
    a. How has your practice of weighing patients changed?
    b. You mentioned [you/the patient] typically raise the topic of weight. Has this changed? [Staff conducting consultations]
2. Was there any training/education you received to implement the Enhanced Service? [All staff]
    a. *If yes:* can you speak to what it was? Did you find it helpful/unhelpful, and in what ways?
    b. *If no*: Do you think it would have been helpful/unhelpful, and in what ways?
    c. How would you like to have been supported to incorporate referrals into your routine care of patients living with obesity?
3. What do you find easy and what do you find difficult about making referrals to weight management programmes? [Staff conducting consultations]
    a. What is your typical approach?
        i. Who usually initiates the conversation on weight?
        ii. *If text messages were sent, ask if this gave the practitioner an easy 'in'*
    b. When you talk to your patients about their weight, do you think it affects your relationship with them?
    c. Have you got a way of speaking about weight that makes it feel comfortable for you and the patient?
        i. How do patients react?
        ii. Do you worry about complaints?

   d. Do you make referrals now for every patient who qualifies under the Enhanced Service?
4. Do you have to work with others in your clinic to fulfil the Enhanced Service? [All staff]
   a. If so, who?
   b. What was that experience like?

Reflexive Monitoring (appraisal, evaluation, how the effects of the intervention are understood)

1. Do you feel that making referrals to weight management programmes is an effective and worthwhile way to manage obesity amongst your patients? If not, why not? If so, how do you know? [All staff]
   a. What about for hypertension, and diabetes?
2. Have you had feedback from the service or from the patients? If so, can you share? [All staff]
3. Do you have any suggestions for improving the promotion and engagement with the Enhanced Service amongst your patients and colleagues? [All staff]
4. Is there anything you would do/do differently now in your consultations to promote the uptake of a referral to a weight management programme? [Staff conducting consultations]
5. The enhanced service pays the practice for the work of referring. How do you feel about that? [Staff conducting consultations]
   a. Would you do this if you were not paid specifically for this?
   b. You are not paid to refer people with other kinds of conditions to services. Why is obesity different? Do you feel differently about it?
6. Do you think the Enhanced Service is a good use of NHS resources? [All staff]
7. Now thinking about the Digital Weight Management Programme specifically; this is the first time the NHS is spending money on tier 2 weight management programmes. Do you think this is a good use of NHS resources? [All staff]
   a. Have any of your patients engaged with the DWMP? If so, can you share any feedback?
8. In our last interview, you said [_xyz_] about obesity management in primary care. Do you still feel the same way? [All staff]

Version B

[Refresh on what the NESI is]

1. Why did your clinic choose TO NOT opt-in, and what do you think about it? [All staff]
2. If you were making the decision, would you have encouraged your clinic to join the scheme? [All staff]
3. Do you think your clinic should have participated? Why or why not? [All staff]

4. Do you feel that you as a [*insert professional role*] can advocate to participate in these programs on behalf of your clinics' patients? [All staff]
5. Do you think the Enhanced Service is a good use of NHS resources? [All staff]
6. Now thinking about the Digital Weight Management Programme specifically; this is the first time the NHS is spending money on tier 2 weight management programmes. Do you think this is a good use of NHS resources? [All staff]

Supplement 2 – NPT Constructs mapped to interview guide

Implementation Outcome	NPT Construct	NPT Sub-construct	Question(s)
Appropriateness<br><br>(i.e. Views on the actions associated with the obesity QOF and relevance to professional roles)	Coherence<br><br>(i.e., Can clinicians and staff make sense of the actions part of the obesity QOF?)	Differentiation	1,3, 7
		Communal Specification	6
Acceptability<br><br>(i.e. general perceptions of the actions suggested by the obesity QOF, and how these perceptions measure up to clinician and staff expectations)		Individual Specification	7
		Internalization	6
Adoption<br><br>(i.e. How have clinician and staff practices changed in terms of personal behaviour? How have their colleagues' practice changed in terms of clinic behaviour?)	Cognitive Participation<br><br>(i.e. Can clinicians and staff get themselves and others involved in implementing the actions suggested by the obesity QOF?)	Initiation	8
		Enrolment	9
		Legitimation	2
		Activation	8
Feasibility<br><br>(i.e. How useful or practical do clinician and staff perceive the actions associated with the QOF to be? What are the barriers and facilitators from their perspective?)	Reflexive Monitoring<br><br>(i.e. Can the intervention be monitored and evaluated? Can effectiveness be judged by clinicians and staff?)	Interactional Workability	12
		Relational Integration	4
		Skill Set Workability	11
		Contextual Integration	10
Penetration<br><br>(i.e. How do the actions suggested by the obesity QOF infiltrate the routine practice of the clinic?)	Collective Action<br><br>(i.e. What needs to be done to make the actions suggested by the obesity QOF work in practice?)	Systematization	5
		Communal Appraisal	14
Sustainability<br><br>(i.e. How are the actions associated with the obesity QOF maintained over time?)		Individual Appraisal	14
		Reconfiguration	13
